# Supplementary material for: Ecotype-specific blockage of tasiARF production by two different RNA viruses in Arabidopsis
Source: PLoS One. 2022 Oct 5;17(10):e0275588. doi: 10.1371/journal.pone.0275588 (PMC9534422; doi:10.1371/journal.pone.0275588)
Supplement: S1 File — The result of a single marker analysis performed by WinQTL Cartographer. (PDF) [file pone.0275588.s007.pdf]

Doc S1. Result of the QTL mapping.

The text below is the output of the Single Marker Association test performed by Windows QTL Cartographer v2.5.

```
This analysis fits the data to the simple linear regression model
              y = b0 + b1 x + e
The results below give the estimates for b0, b1 and the F statistic for each marker.

We are interested in whether the marker is linked to a QTL. We test this idea by
determining if b1 is significantly different from zero. The F statistic compares the
hypothesis H0: b1 = 0 to an alternative H1: b1 not 0. The pr(F) is a measure of how much
support there is for H0. A smaller pr(F)indicates less support for H0 and thus more
support for H1. Significance at the 5%, 1%, 0.1% and 0.01% levels are indicated by *, **,
*** and ****, respectively.

Note that our Likelihood ratio test statistic compares two nested hypotheses and is two
times the negative natural log of the ratio of the likelihoods. For example, assume that
hypothesis H0 is nested within H1 and that they have likelihoods L0 and L1 respectively.
Then, the "Likelihood Ratio Test Statistic" is -2ln(L0/L1).

-t    1 is the number of trait being analyzed (Leaf_curvature).
-----------------------------------------------------------------------------
-----------------------------------------------------------------------------

Chrom.  Marker     b0         b1      -2ln(L0/L1)   F(1,n-2)      pr(F)
-----------------------------------------------------------------------------
01         1      1.770     -0.312          2.499      2.453   0.124992992
01         2      1.745     -0.216          1.144      1.105   0.299252748
01         3      1.744     -0.182          0.788      0.758   0.389077549
01         4      1.731     -0.198          0.907      0.874   0.355370391
01         5      1.731     -0.198          0.907      0.874   0.355370391
01         6      1.777     -0.044          0.045      0.043   0.837594677
01         7      1.697     -0.236          1.204      1.164   0.286900463
01         8      1.713     -0.239          1.271      1.230   0.273830940
01         9      1.745     -0.109          0.243      0.232   0.632430774
01        10      1.800      0.029          0.018      0.017   0.896459845
01        11      1.800      0.029          0.018      0.017   0.896459845
01        12      1.788     -0.058          0.082      0.078   0.780854176
01        13      1.734      0.163          0.591      0.567   0.455596341
01        14      1.817     -0.055          0.059      0.057   0.813288173
01        15      1.859     -0.185          0.728      0.700   0.407657888
01        16      1.771      0.057          0.071      0.068   0.795461093
01        17      1.790      0.002          0.000      0.000   0.991229893
01        18      1.797     -0.037          0.033      0.032   0.859821464
01        19      1.782     -0.132          0.438      0.419   0.520892098
01        20      1.781     -0.059          0.085      0.082   0.776625650
01        21      1.781     -0.059          0.085      0.082   0.776625650
01        22      1.761     -0.099          0.226      0.216   0.644589183

02         1      1.784      0.068          0.114      0.109   0.743282413
02         2      1.789      0.075          0.141      0.135   0.715365311
02         3      1.791      0.095          0.225      0.215   0.645529435
02         4      1.786      0.105          0.275      0.263   0.610870258
02         5      1.781      0.201          1.006      0.971   0.330261311
02         6      1.795      0.085          0.180      0.172   0.680196767
02         7      1.748      0.263          1.647      1.601   0.212926911
02         8      1.729      0.444          5.046      5.105   0.029230281 *
02         9      1.695      0.457          5.099      5.162   0.028397602 *
02        10      1.722      0.492          6.302      6.472   0.014825820 *
02        11      1.727      0.393          3.988      3.985   0.052591281
02        12      1.736      0.473          5.967      6.103   0.017742784 *
02        13      1.736      0.473          5.967      6.103   0.017742784 *
02        14      1.739      0.446          5.021      5.078   0.029645023 * FIL

03         1      1.791      0.333          2.836      2.795   0.102160522
03         2      1.825      0.364          3.377      3.350   0.074478170
03         3      1.825      0.364          3.377      3.350   0.074478170
03         4      1.827      0.261          1.682      1.635   0.208162635
```

03	5	1.813	0.105	0.256	0.244	0.623724045	
03	6	1.793	0.018	0.008	0.008	0.931200234	
03	7	1.794	0.158	0.636	0.611	0.439036018	
03	8	1.834	0.376	3.682	3.665	0.062552198	
03	9	1.741	0.425	4.770	4.810	0.034016468	*
03	10	1.772	0.272	1.900	1.852	0.181000452	
03	11	1.788	0.121	0.373	0.357	0.553374761	
03	12	1.788	0.121	0.373	0.357	0.553374761	
03	13	1.794	0.141	0.484	0.464	0.499558456	
03	14	1.792	0.065	0.107	0.102	0.751219392	
03	15	1.807	0.140	0.494	0.474	0.495045038	
03	16	1.802	0.093	0.217	0.208	0.651006256	
04	1	1.763	0.237	1.423	1.379	0.247021667	
04	2	1.800	0.391	4.042	4.041	0.051014817	
04	3	1.811	0.289	2.157	2.109	0.154038390	
04	4	1.782	0.354	3.288	3.258	0.078419297	
04	5	1.747	0.378	3.727	3.712	0.060971184	
04	6	1.734	0.346	3.045	3.009	0.090337667	
04	7	1.734	0.346	3.045	3.009	0.090337667	
04	8	1.742	0.298	2.241	2.194	0.146202841	
04	9	1.785	0.085	0.181	0.173	0.679448484	
04	10	1.787	0.168	0.717	0.690	0.411126745	
04	11	1.791	-0.024	0.014	0.013	0.908560495	
04	12	1.781	0.138	0.459	0.440	0.510793144	
04	13	1.731	0.320	2.253	2.206	0.145140881	
04	14	1.775	0.222	1.144	1.105	0.299305012	
04	15	1.756	0.212	1.070	1.033	0.315302965	
05	1	1.788	0.038	0.036	0.035	0.853024294	
05	2	1.769	0.190	0.907	0.874	0.355280557	
05	3	1.758	0.202	1.019	0.983	0.327293004	
05	4	1.746	0.146	0.496	0.475	0.494492503	
05	5	1.757	0.132	0.413	0.396	0.532602700	
05	6	1.759	0.225	1.242	1.201	0.279467028	
05	7	1.780	0.116	0.332	0.318	0.576102969	
05	8	1.789	0.075	0.142	0.135	0.714693624	
05	9	1.802	0.252	1.601	1.555	0.219472512	
05	10	1.837	0.334	2.787	2.746	0.105149777	
05	11	1.831	0.345	2.931	2.892	0.096611818	
05	12	1.823	0.282	2.028	1.980	0.166916274	
05	13	1.819	0.383	3.832	3.822	0.057437069	
05	14	1.791	0.595	9.785	10.477	0.002395174	**
05	15	1.791	0.595	9.785	10.477	0.002395174	**
05	16	1.781	0.400	4.257	4.266	0.045234799	*
05	17	1.776	0.633	11.553	12.637	0.000969425	***
05	18	1.776	0.633	11.553	12.637	0.000969425	*** ARF4
05	19	1.749	0.599	10.132	10.894	0.002003949	**
05	20	1.831	0.436	4.933	4.984	0.031106667	*

#EndTrait 1
