# Supplementary material for: Ecotype-specific blockage of tasiARF production by two different RNA viruses in Arabidopsis
Source: PLoS One. 2022 Oct 5;17(10):e0275588. doi: 10.1371/journal.pone.0275588 (PMC9534422; doi:10.1371/journal.pone.0275588)
Supplement: S2 File — (PDF) [file pone.0275588.s008.pdf]

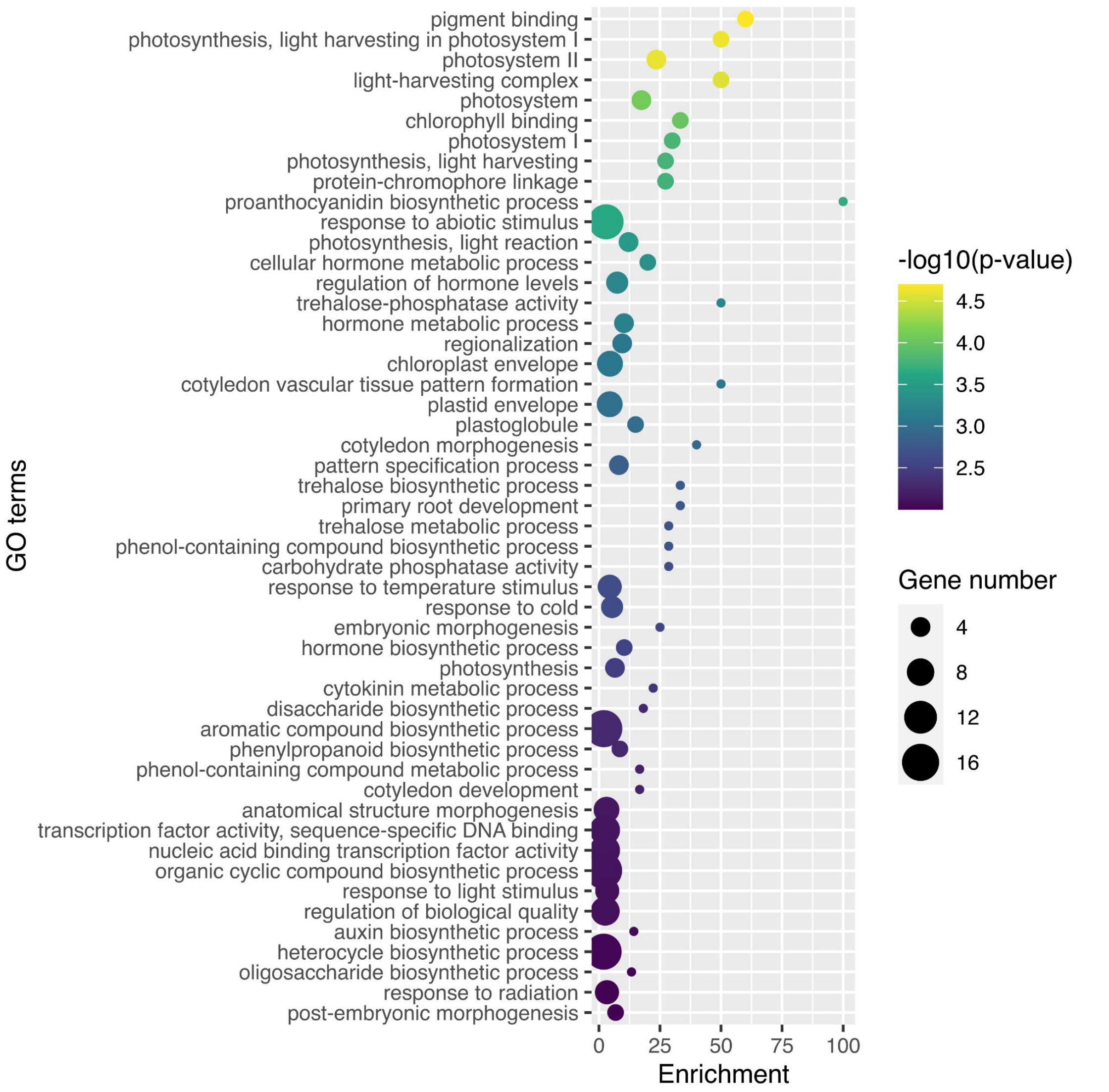

Fig S1. GO term enrichment analysis of the downregulated, potential vasiRNA target genes.

The common DEGs of the TVCV and TuMV-infected Bur plants (orange and blue colored sets in Fig 4) that are predicted to be regulated by DESs (differentially upregulated virus-activated sRNAs, vasiRNAs) were subjected to GO-term enrichment analysis using the PlantRegMap server with the default settings. Only terms in the Biological Process category are shown, the results of the full analysis can be found in Table S3e. Enrichment was calculated by dividing the observed number of genes in a particular category by the expected number of genes in that category.

Fig S2. Amino acid alignment of ARF4, FIL, AGO1, AGO2, and AGO7 in 19 different ecotypes. Alignments were created with Clustalo and colored by % identity using Jalview. The Col and Bur sequences are highlighted/bracketed.

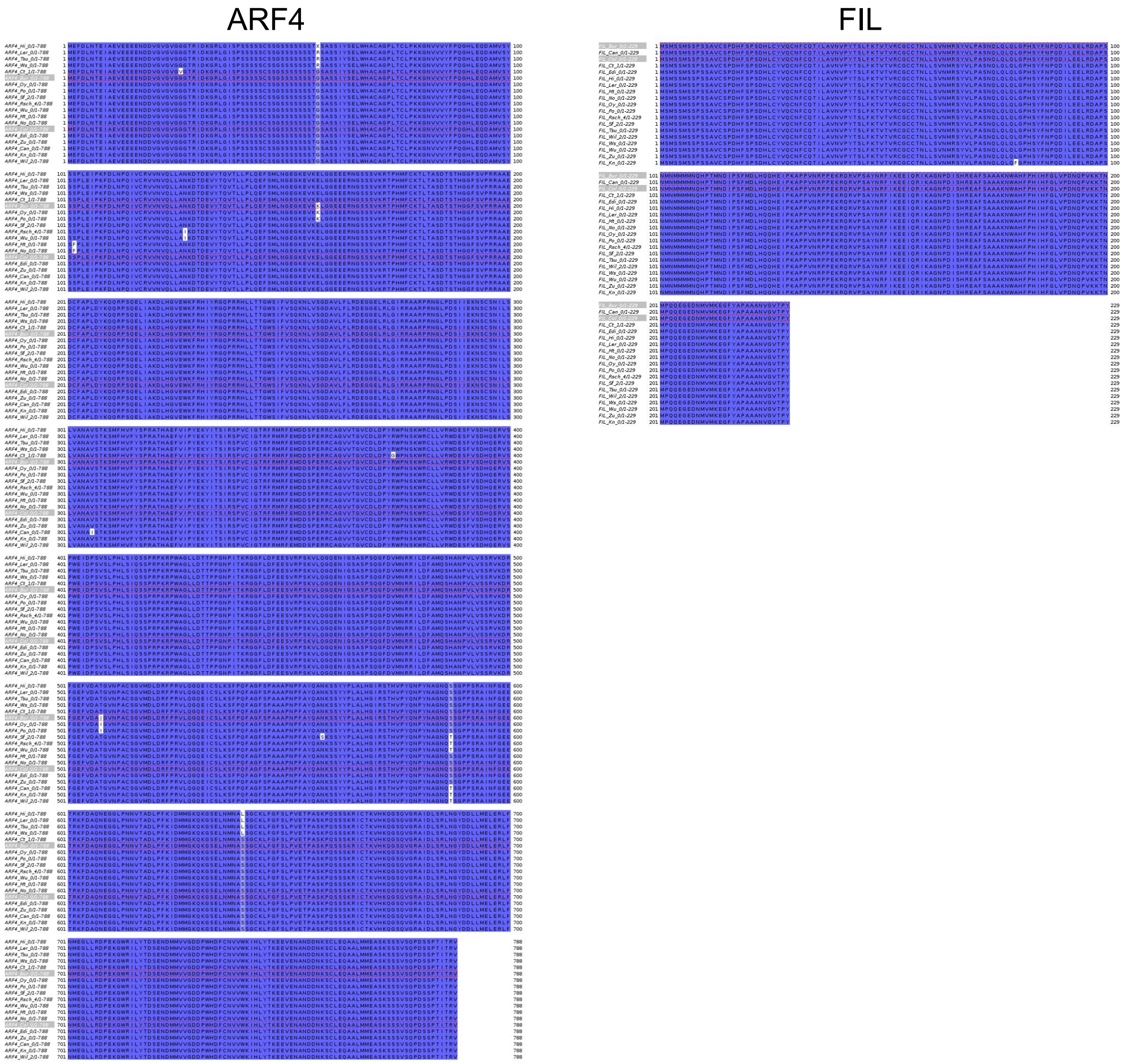

Figure continues on next page

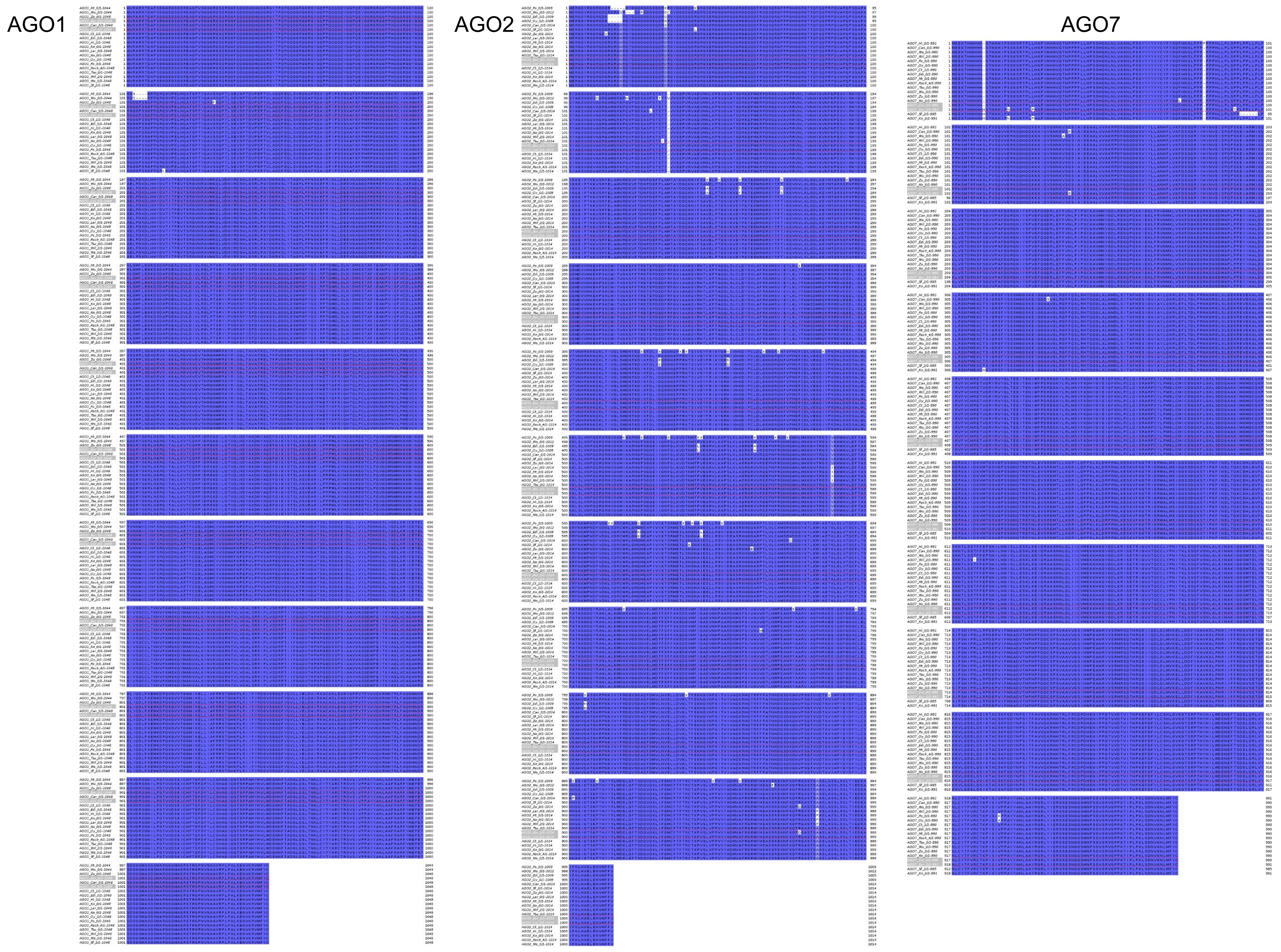
AGO1
AGO2
AGO7
